# Gut Microbiota, Metabolic Disorders and Breast Cancer: Could Berberine Turn Out to Be a Transversal Nutraceutical Tool? A Narrative Analysis

**DOI:** 10.3390/ijms232012538

**Published:** 2022-10-19

**Authors:** Massimiliano Cazzaniga, Giordano Bruno Zonzini, Francesco Di Pierro, Sara Moricoli, Alexander Bertuccioli

**Affiliations:** 1 Scientific & Research Department, Velleja Research, 20125 Milano, Italy; 2 Department of Biomolecular Sciences, University of Urbino Carlo Bo, 61029 Urbino, Italy; 3 Digestive Endoscopy Unit and Gastroenterology, Fondazione Poliambulanza, 25124 Brescia, Italy; 4 AIFeM, 48100 Ravenna, Italy

**Keywords:** microbiota, dyslipidemia, estroboloma, breast cancer

## Abstract

Metabolic disorders, mainly characterized as the marked alteration of the lipid and carbohydrate profile, in addition to the clinical presence of the direct consequences of these alterations, are pathological conditions that have considerably increased in prevalence in recent years. They are directly linked to the onset of various pathologies, including cancer, particularly breast cancer, and are hormone-responsive. Alongside the known conditions responsible for this scenario, such as nutrition and lifestyle in general, the importance of both the colonic microbiota and the various organs and systems is becoming increasingly evident. In fact, it is now evident that microbial dysbiosis plays a fundamental role in the onset of these metabolic disorders, and therefore how these conditions are indirectly responsible for the onset and progression of neoplasms. Indirect mechanisms such as an altered Firmicutes/Bacteroidetes ratio; the formation of metabolites such as short-chain fatty acids (SCFAs), in particular, butyrate, which is capable of acting as a tumor suppressor; and the glucuronidase activity of estroboloma (bacteria responsible for estrogen metabolism) are just some of the most important mechanisms that contribute to the history of breast cancer. It is therefore understandable that in clinical terms, it is essential to associate the modulation of metabolic disorders and the microbial conditions that contribute to generating them with common therapies, preferably using compounds and solutions that are effective and acceptable for the patient without side effects. Nutraceuticals such as berberine (active both in metabolic scenarios and in the microbiota) and interventions modulating the microbial structure such as the use of probiotics and prebiotics seem to be ideal solutions for these preventive and no-longer-ignorable strategies in the light of numerous data now present in the literature.

## 1. Introduction

As with many types of neoplasms, breast tumors do not appear as pathologies originating from a single cause that can alone explain the onset of the tumor, but rather are recognized as having a multifactorial genesis. It is now widely demonstrated that among the various modifiable risk factors for breast cancer, there are some important elements that we can roughly classify as metabolic disorders. These include a whole series of hormonal, inflammatory and metabolic conditions which, if not adequately treated and controlled, exponentially increase the risk of becoming ill and/or not responding to cancer therapies [1]. Until now, the attention of the medical profession has mainly focused on the modification, often pharmacological, of various conditions (e.g., obesity and hyperglycemia above all) and attempts to change the lifestyles responsible for the onset of the same. However, research in this field has recently begun to understand how the microbiota (particularly the colonial one) has a fundamental influence in this scenario, being able to condition the generation of these metabolic disorders and therefore impact the onset and progression of these neoplasms [2]. There are many ways in which the composition and activity of the gut microbiota act on mammary carcinogenesis, and in this review, we will try to highlight the pathophysiological mechanisms underlying this influence and the possible clinical intervention strategies.

## 2. Metabolic Disorders as a Risk Factor for Breast Cancer

Numerous studies have highlighted how the risk factors acquired and related to an incorrect lifestyle, be they reproductive or dietary, are responsible for about one-third of all cancers, and how the prevalence of what we define as metabolic disorders (meaning the presence of obesity, overweight and/or metabolic syndrome, diabetes and/or type 2 diabetes mellitus, and even single alterations in the lipid and carbohydrate profile of a subject) has reached 35% [3], in this way exceeding the importance of other more commonly recognized and immediate risk factors such as cigarette smoking. These data are referring to a general oncological scenario do not change when compared with female tumors and in particular with mammary ones. One of the most authoritative reviews on the subject published in 2016 [4] certified the relationship between obesity and the incidence/mortality of various female cancers, and a confirmation of the impact of obesity and overweight on the outcome of breast disease was also reported in a secondary analysis of the WHI study [5] and in an interesting work from 2018, where it was evident that any change in weight (both gains and losses) during chemotherapy reduces the effectiveness of the latter [6]. This is also confirmed by linking metabolic alterations of the glucose profile with an increase in incidence and mortality both in the presence of diabetes (type 1 and 2) and with the more nuanced conditions of hyperglycemia and hyperinsulinemia [7].

### Mechanisms That Bind Metabolic Disorders and Carcinogenesis

**Figure 1:**
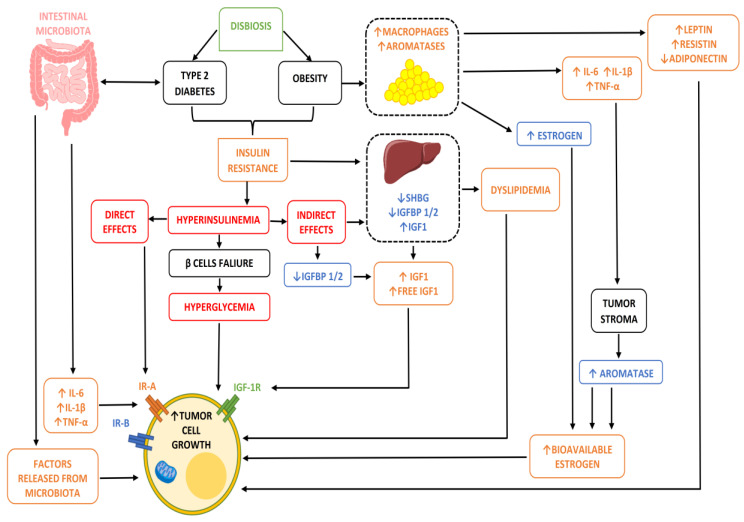
Correlation between more frequent metabolic disorders and tumor growth. The microbiota favoring or disfavoring the onset of metabolic disorders ends up impacting the onset and progression of neoplastic disease.

The biological mechanisms that correlate metabolic states and the onset or progression of breast cancer are multiple, complex and still not fully clarified, particularly regarding their sequence. However, what is certain is that very important and well-established hormonal, inflammatory, growth and microbial factors come into play in this relationship. Figure 1 below covers most of these factors (at least the most important and frequent) by placing them in correlation with each other and highlighting the multidisciplinary nature of the situation and the influence that the various conditions have on the final event. Figure 1 only summarizes the role played by the most frequent metabolic disorders, to which all of the mechanisms already known in the pathogenesis of breast cancers are added, such as genetically determined ones.

In detail, we can observe that:

(1) In conditions of obesity or overweight, our adipocytes, due to their hypertrophy, undergo a state of hypoxia and necrosis and a consequent alteration of their metabolic activities. In particular, the alteration of the production of some adipocytokines such as adiponectin and leptin, and above all their relationship, is implemented, ultimately increasing the action of leptin which, being an element with proliferative and angiogenetic capacity, is potentially dangerous when present in high quantities and for a long time in our body [8]. Furthermore, this condition of adipocyte suffering also has the non-negligible effect of increasing the inflammatory response by causing chronic inflammation through the recall of immune cells (lymphocytes and macrophages) and the consequent response by cytokines. Clearly, it is not negligible that in these conditions of obesity/overweight, an increase in the adipose content ultimately also means a greater share of active aromatase enzyme and consequently a greater share of free estrogenic hormones potentially stimulating and proliferating on the mammary gland or on the other hormonally sensitive areas.
(2) In the presence of obesity, MS or diabetes, insulin resistance (IR) is often established. This ultimately leads to hyperglycemia (glucose not being sequestered at the cellular level, and rather remaining in circulation) and hyperinsulinemia (the pancreas, feeling that hyperglycemia is present, is stimulated to produce new insulin), and these two situations are potentially one oncological bomb, being capable of intervening both directly and/or indirectly in the transformation and progression of a breast cell in oncology [9].
(3) But why are hyperinsulinemia and hyperglycemia oncogenetics? That is, why do they stimulate the onset and progression of cancer cells? Insulin is a growth factor, and by its nature it stimulates cell growth and proliferation by acting either directly on the cell using its own receptors or by increasing the share of insulin-growth factor (IGF) thanks to the reduction in the number proteins which, by binding to this element, remain inactive (IGFBPs) [9]. In addition, insulin and hyperinsulinemia also have metabolic action by altering, for example, the adiponectin/leptin ratio seen above, increasing aromatase activity, and stimulating angiogenic activity (i.e., the ability of cancer cells to build their own blood vessels in order to guarantee the sustenance necessary to survive and grow).
(4) Unlike insulin, blood sugar does not have any real oncological action, but rather indirectly favors the growth of a tumor, providing an ideal environment for its survival and growth. This occurs due to the metabolic reprogramming or Warburg effect. In practice, cancer cells guarantee themselves the energy to survive in a different way to normal ones, favoring the process of anaerobic glycolysis compared to that of oxidative phosphorylation (OXPHOS). The process of glycolysis is strictly interconnected with the amount of glucose available, as it is less efficient from an energy production point of view than phosphorylation and is therefore facilitated by the condition of hyperglycemia. Authoritative studies and meta-analyses have already highlighted and confirmed the relationship between IR/hyperglycemia and female tumors [10].
(5) Alterations in the lipid profile often accompany dysmetabolic conditions, and hypercholesterolemia is now a recognized risk factor for various female cancers and in particular for breast cancer. Biologically speaking, hypercholesterolemia is now a recognized risk factor for cancer by virtue of both direct and indirect action on target tissues for hormonal activity. In fact, as we know, cholesterol is transformed in our body into estrogen hormones (this mechanism is all the more important when the aromatase enzyme is involved, as happens after menopause and as we have seen abundantly represented in the presence of excess adipose tissue) which, as we know, have a stimulatory and proliferative action on sensitive tissues. Not only that, but cholesterol in large quantities is also transformed by the enzyme CYP27A1 into its active metabolite called 27 hydroxycholesterol (27HC), which has a chemical structure very similar to that of estrogen and is therefore also capable (although it is not a real hormone) of binding to hormone receptors, igniting all of the proliferative pathways usually associated with this situation downstream [11]. These biological mechanisms therefore lead to hypercholesterolemia, which is a condition directly and indirectly affecting the incidence (little) and prognosis (a lot) of oncological disease thanks to its being a source of hormonal stimulation. This is further burdened by the fact that some of the key treatments involved in post-surgical adjuvant therapy, such as aromatase inhibitors (which aim at the total elimination of circulating estrogen hormones, therefore being substantially less effective in the presence of hypercholesterolemia), have the increase in lipid values among their main side effects, and in particular those of cholesterol (especially letrozole and anastrazole).

## 3. How Does the Gut Microbiota Determine Metabolic Disorders and Increase Tumor Risk?

In recent years, alongside these consolidated risk factors, the importance of the microbiota and, in particular, the impact that some “dysbiotic” conditions can have on the onset and progression of cancer have become clear, and no longer negligible. In particular, the gut microbiota, or rather some of its composition or equilibrium alterations, is able to influence the onset of metabolic and hormonal conditions capable of indirectly influencing (as already highlighted) the carcinogenesis and progression of cancer. Specifically, it is demonstrated that conditions such as obesity, insulin resistance, chronic inflammation and hyperestrogenism, which are closely related to the genesis of numerous types of cancer, such as those of the female reproductive system, are also largely determined from states of dysbiosis of the intestinal microbiota [2]. The mechanisms through which this happens are various and complex. Among the most important, we highlight the following:

(1) Firmicutes/Bacteroidetes ratio;
(2) Production and use of butyrate as an oncosuppressor;
(3) Hormonal entero-hepatic circle.

### 3.1. Firmicutes/Bacteroidetes Ratio

**Figure 2:**
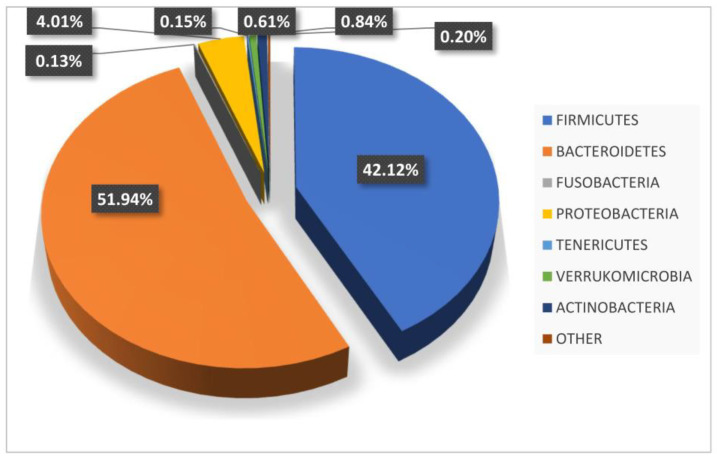
Percentage composition of the microbiota in a eubiotic subject.

In general, as we all know, the analysis of the bacterial phyla of the gut microbiota demonstrates, in adults, the presence of two primarily dominant groups which together constitute approximately 95% of the microbial populations. These are known as the Bacteroidetes and Firmicutes. The remaining about 5% is mainly attributable to the phyla Proteobacteria, Actinobacteria, Tenericutes, Verrucomicrobia and Fusobacteria (Figure 2).

Bacteroidetes, present on average at 45–55%, are all Gram-negative bacteria. It is believed that for analytical values around 50–60%, they confer a protective role against the manifestation of obesity, and that their decrease, in favor of the phylum Firmicutes, can instead predispose more to the appearance of an obese phenotype [12]. On the contrary, when particularly high, with values higher than 70–75%, it seems that they expose the host to a risk of diabetes, and perhaps to other inflammatory diseases, especially in the presence of a high percentage of Proteobacteria. Firmicutes, even if present at a percentage lower than Bacteroidetes, are believed to make up about 40–45% of the entire fecal microbiota under normal conditions. They are by far the most heterogeneous and vast phylum of all. This phylum is mainly made up of (but not limited to) Gram-positive bacteria, and very high relative values seem to predispose to an obese phenotype due to an increased ability to extract calories from food. In consideration of what has been described so far, it appears clear that the intestinal microbiota, and in particular the relationship between these two dominant phyla, is an important environmental factor capable of influencing the energy balance of the host, the appearance or control of some important metabolic disorders and, consequently, their impact on the incidence and outcome of tumor pathology.

(1) The suppression of the expression of a lipoprotein with inhibitory functions of intestinal lipase (LPL), the so-called fasting-induced adipose factor (FIAF), with a consequent increase in the extraction capacity of fatty acids from postprandial fat particles and an increase in the accumulation of fat inside the adipocytes,
(2) The increased ability to form short-chain fatty acids (SCFAs), in particular butyrate, by some bacterial populations (clostridiales) with consequent accumulation of calories in the form of body fat.
(3) The increase in the permeation of lipopolysaccharides (LPS) due to a decrease in the share of SCFAs in the event of a decrease in clostridiales increases the release of inflammatory cytokines, primarily TNF-α, through a mechanism of activation of Toll-like receptor-4 (TLR4), its specific receptor, with an increase in peripheral inflammatory phenomena and the possible development of hepato-steatosis, insulin resistance, omental inflammation, metabolic syndrome and diabetes

These are all conditions through which an alteration in the F/B ratio contributes to increasing cancer risk, indirectly increasing the onset of metabolic disorders.

### 3.2. Production and Use of Butyrate as Oncosuppressor

**Figure 3:**
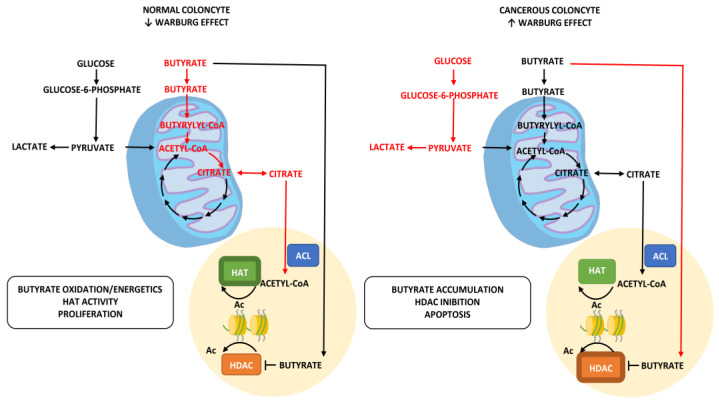
Oncosuppressive role of butyrate. Under normal conditions, butyrate is mainly used as an energy producer by cells. In those tumors, the energy is obtained in particular from glucose (Warburg effect) and the excess butyrate is sent to the nucleus where, being a natural acetylator, it contributes to a tumor antiproliferative action.

Butyrate (or butyric acid), generated thanks to the action of some bacterial groups (in particular clostridiales) in the fermentation of non-digestible carbohydrates (fibers, FOS, GOS inulin, etc.) which come into direct contact with the gut, plays, as already mentioned, an important metabolic role. However, it also has an important acetylation capacity, making it useful in counteracting some oncological phenomena such as de-acetylation and/or DNA methylation that promote and stimulate pathways such as cell proliferation [13] (Figure 3). It is therefore understood how the production capacity of an adequate amount of butyrate can be useful in countering potentially pathological pathways and therefore how an adequate amount or bacterial balance is fundamental for this purpose. Specifically, it should be remembered how cancer cells, due to their metabolic reprogramming (Warburg effect), consume a greater share of glucose to produce energy than non-pathological cells. This process, also exploited in the diagnosis and in oncological treatments (PET and ketogenic diet, for example), will have as a side effect a quota of butyrate produced in excess that will not be used metabolically but diverted to the nuclear level, where it will act as an acetylator (anti-proliferator). In practice, it works similarly to drugs that are often used for their ability to block the de-acetylation of histones (histone deacetylase inhibitors). Obviously, this “beneficial and protective” effect of butyrate is conditioned by its adequate production, and therefore by the presence or absence of an adequate number of bacteria in charge of it. Dysbiosis or conditions that decrease, for example, the share of Firmicutes (clostridiales) end up weakening this protective pathway, consequently increasing the tumor risk.

### 3.3. Hormonal Entero-Hepatic Circle

**Figure 4:**
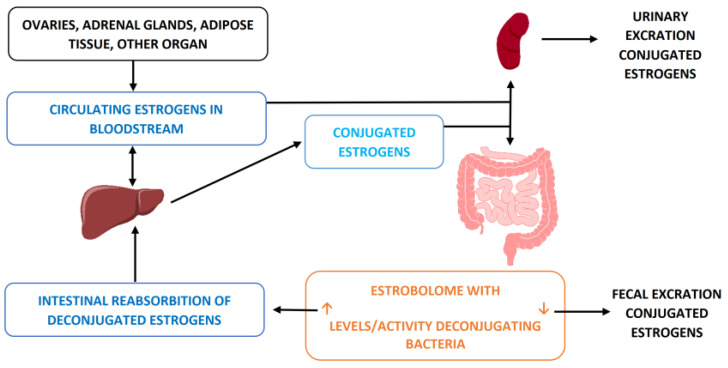
Entero-hepatic circle of estrogens. Some bacteria endowed with b-glucuronidase activity deconjugate estrogens by reintroducing them into the circulation and thus increasing their stimulatory action on the receptors and therefore also the oncological potential, in particular, for hormone-dependent tumors, such as most mammary ones.

This condition can be summarized as a greater share of circulating estrogens (in addition to those possibly increased by the presence of obesity and by the greater aromatase activity) resulting from the action of the gut microbiota and is an inherent risk factor for all hormone-dependent oncological diseases such as mammary and endometrial cancer. To understand how this dangerous scenario arises, it is necessary to remember how normally circulating estrogens, once their function has been performed, are conjugated in the liver in order to be eliminated via the urinary or fecal route. Clearly, this last portion, passing through the intestine, comes into contact with the gut microbiota which, under certain conditions, can modify this process. In particular, the presence of a greater share of bacteria with b-glucoronidase activity can, through this action (determined by an enzyme they produce called b-glucuronidase), deconjugate estrogens, making them active again and reintroducing them into the circulation [14] (Figure 4). This estrogenic surplus circulating is obviously a risk factor for all of the target organs (rich in estrogen receptors).

## 4. Possibility of Intervention Treatment of Metabolic Disorders: Expand Therapeutic Background

From what has been described so far, it is quite evident that the metabolic disorders and the alterations of the intestinal microbiota that favor and amplify them are non-negligible risk factors for mammary tumors, and therefore, it is essential to include the prevention and treatment of these conditions in normal clinical oncological, as well as cardiovascular, practice. It is equally clear how the treatment can be applied to the two levels described—that is, both to the metabolic conditions present and to the conditions, in particular those of microbial dysbiosis, that cause them.

There are many compounds (old and new generation) that, within the literature, have already shown the ability to interfere with these biological parameters (and therefore to interfere with a whole series of problems, particularly cardiovascular), but only some of these have also been studied in the sense of oncology, highlighting, coincidentally, extremely interesting results. Among these, the main ones, given that we have basically talked about alterations in the carbohydrate and lipid profile, are metformin and statins.

There are now numerous data in the literature proving the effectiveness of these treatments in the oncology field. Many meta-analyses have now highlighted how the use of therapeutic strategies of this type decreases the incidence and improves the prognosis of various types of cancers, including breast cancers. Although, it should be logical and consequential to use these treatments in all our patients with oncological pathology (therapy) or in those considered at increased risk of developing it (prevention), some obstacles make this application difficult. Among the most common, unfortunately, is the lack of medical sensitivity on the subject, along with objective problems such as unacceptability by patients, fear and/or the presence of side effects, and the presence of borderline or only slightly altered metabolic values that recommend a non-therapeutic approach (food and lifestyle) that is often destined to fail.

Hence, the need to find, know and use alternative/synergistic (complementary) compounds capable of guaranteeing the same activity and efficacy as conventional ones (alone or in association with them), canceling or mitigating their side effects has arisen. There are many compounds currently under study that show these properties and characteristics and that bode well for their future use also in the oncology field [15]. Some of these, however, already enjoy unequivocal data in the literature, making their current scarce use in oncological terms incomprehensible. The main compound in this sense is berberine, which boasts more than 7000 publications on PubMed, with about 1300 of these relating to its anticancer activity [16]. This compound is suitable and optimal, as it is active both on metabolic disorders and on the microbiota that generates them, thus providing a wide and complete coverage in preventive and therapeutic terms.

### 4.1. Berberine on Metabolic Factors

Berberine is an alkaloid extracted from the root of plants, mainly of oriental origin, of the genus Berberis (BBR-*Berberis aristata* and other spp.). It is effective in controlling cholesterol linked to LDL lipoproteins, but also in favorably influencing the overall lipid profile (therefore including plasma levels of triglycerides and HDL cholesterol) as well as blood sugar [17,18,19,20,21,22,23,24]. There are now many points of data in the literature that confirm these therapeutic qualities of the compound in the face of a significantly better safety and acceptability profile than conventional therapies [25,26]. The action on the lipid profile of BBR obviously takes place in a lipid-lowering sense, and mainly on LDL. Its action takes place mainly in the liver, where the BBR at this level is able to increase the LDL receptors (LDLR) in two ways—a direct way, by facilitating the hepatic construction of the receptor with a stabilizing action on the LDLR mRNA, and an indirect way, by decreasing the level of subtilisin/kexin protein convertase type 9 (PCSK9), whose action usually results in the decreased efficiency of the LDL receptor.

However, what makes this compound unique and essential in this field is its incredible ability to modulate not only the lipid profile but also act simultaneously on the carbohydrate profile [24,27]. The glycemic control mechanism by berberine is also complex, and correlates both with the ability of this molecule to reduce intestinal absorption of glucose and with the ability to increase muscle and hepatic uptake of glucose itself [21,22]. However, the main mechanism that makes it absolutely comparable both in terms of efficacy and safety to pharmacological compounds such as metformin is making the cells directly more sensitive to the action of insulin with the activation of AMPK (remember that the BBR acts at the mitochondrial level similar to metformin, on the complex I of the mitochondrial respiratory chain, blocking its action and therefore favoring the activation of AMPK) [23]. This effect of insulin sensitization has, as a direct consequence, a decrease in blood sugar and circulating insulin and their direct carcinogenic properties.

### 4.2. Berberine on Microbiota

In consideration of the fact that, as highlighted, the metabolic disorders that correlate with the incidence and progression of tumors are often associated with conditions of dysbiosis of the microbiota, it is understandable that an effective preventive or therapeutic treatment cannot be separated from the control of this variable. We have seen how numerous and important scenarios induced by alterations to the bacteria of the gut microbiota are able to interfere with various pathophysiological processes that lead to the formation and progression of numerous pathologies, including tumors [28,29,30,31,32], and consequently, it is no longer acceptable to disregard any possible ways to improve our ability to modulate this scenario as well [33].

**Figure 5:**
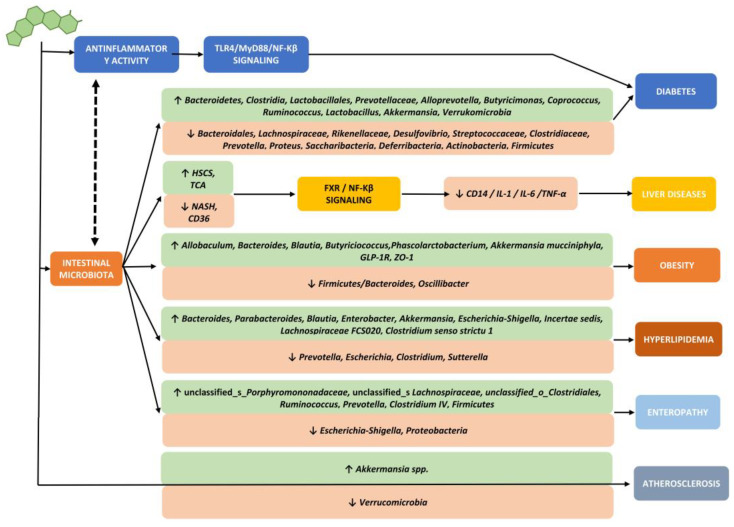
Main actions of berberine on the microbiota and metabolic diseases.

Incredibly, in addition to directly modulating metabolic parameters, the BBR seems capable of interfering and intervening in this area as well, having also shown an ability to modulate the biodiversity and quality of the intestinal microbiota in an eubiotic sense. There are many mechanisms (some described in the first half of the last century) by which this extraordinary compound modulates the microbiota [34,35,36,37,38,39,40,41,42,43], but the most important can be summarized in Figure 5, and in particular, its ability to increase the bacterial share relating to *Akkermansia muciniphila* (AM) [44].

Furthermore, in considering the potential actions of berberine, we should underline its role, direct and mediated by the action on the microbiota, in the modulation of sub-clinical inflammation, a factor of considerable importance in the pathogenesis and natural history of numerous pathologies, including oncological ones. As demonstrated by numerous reviews in the literature, the intestinal microbiota can promote sub-clinical inflammation, relevant in numerous pathologies, with elements such as impaired intestinal permeability, production of lipopolysaccharides (LPS) and bacterial metabolites [44,45,46,47,48,49,50]. These factors are also relevant in the oncological field [51], confirming the transversal applicative potential of Berberian described in Figure 5.

**Figure 6:**
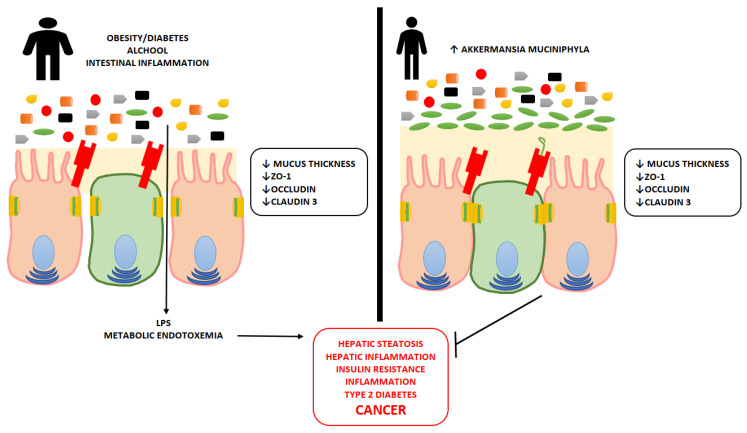
Action of Akkermansia mucciniphyla on mucus production and protection from pathogenic and inflammatory bacteria.

AM is responsible for the thickness of the mucus layer of the GUT. The amount of this bacterium affects body weight and metabolic disorders in the sense that the amount is inversely proportional to the presence or absence of conditions such as obesity or diabetes. In fact, when the AM is poorly expressed, the thickness of the intestinal mucus decreases and consequently the protective thickness of the intestinal membrane becomes small, favoring its permeability. This involves the passage of substances such as lipopolysaccharides (LPS) into the membrane and the phenomenon of endotoxemia with consequent low-grade inflammation, obesity and insulin resistance. BBR acts by increasing the amount of AM, which, by increasing the thickness of the mucus, decreases the permeability of the membrane by reducing the pathogenetic activity that its increase entails, including that of the ignition of oncologically active pathways such as inflammation and proliferation [52,53] (Figure 6).

Finally, it should be noted that, as often happens in the use of nutraceuticals and/or phytotherapics, despite the main problem of inclusion in clinical practice, caused by the poor bioavailability of the compounds and the low quantity of drugs that reach their destination, for BBR, this problem has now been overcome by associating it with silymarin (milk thistle extract) used as a herbal bioenhancer and inhibitor of the major glycoprotein P (gp-P) responsible for poor cellular absorption [54], or by producing it in phytosomal forms such as a recent commercially available formulation [55,56,57].

### 4.3. Bacterial Therapies: Integrate the Potential of Berberine

A still very recent topic with very “young” clinical data is certainly that of bacterial therapies—that is, the use of compounds such as prebiotics and probiotics to modulate the microbial composition and interfere with the history of numerous diseases [58,59,60,61]. Although this is a fairly well-known field, for example in relation to that of pediatric and/or intestinal diseases, little is still known about the effects and the ability to manipulate our microbiota in a metabolic and consequently (as already highlighted) neoplastic sense. In these so-called bacterial therapies, we must necessarily distinguish and specify some definitions in order to then understand the rationale for their use. When we talk about probiotics, we are referring to live and selected microorganisms that, subject to their resistance, rooting and colonization capacity, are able to modify the microbial composition of the recipient and to modulate it according to the purpose to be achieved. Prebiotics, on the other hand, are substances added to the diet or administration which do not perform a nutritional action and are not digestible (fiber, FOS, GOS, inulin) but positively influence the host by selectively stimulating the growth and/or activity of a selected and limited number of native bacterial species. They are, therefore, compounds that favor the conditions for a certain probiotic species (for example, Bifidobacteria) to proliferate. Precisely for this reason, probiotics and prebiotics are often used in combination (there are numerous commercial products already structured in this way), i.e., live products with a substrate suitable for their growth, and are called symbiotics (e.g., Bifidobacteria + FOS). There are numerous mechanisms of action involved in bacterial therapies and, consequently, the functions exercised by a treatment of this type. The mechanisms of action can generally be summarized in the ability to re-establish intestinal eubiosis, to increase the production of SCFAs, and to stimulate anti-inflammatory and immune actions. Consequently, in terms of clinical activity, we can now safely confirm the ability of bacterial treatments to modulate various metabolic processes, and consequently to act indirectly on the various pathologies related to them, including tumors. A recent review [62] collected some of the most important clinical studies (in patients) on the subject, demonstrating the positive effect of bacterial therapies on specific conditions such as obesity, insulin resistance and T2DM, among others. The potential of the tools that can be used in bacterial therapy, as just described, is very interesting. As these are relatively recent applications, caution is required in the evaluation of clinical applications, with particular attention paid to the result of future research on the matter.

## 5. Conclusions

In this article, we discussed the role of berberine as a transversal nutritional tool for the management and add-on therapy of metabolic aspects and those related to the microbiota in breast cancer. The efficacy described for berberine in the management of metabolic aspects, the ability to modulate the intestinal microbiota, the considerable safety of use and the ease of intake represent significant strengths. These factors contribute to the correction of negative parameters not only in an oncological but also in a cardiovascular sense. Elements of weakness are constituted primarily by the future need to demonstrate not so much the ability of berberine to modulate the microbiota, but to do so with a consequent impact on maternal carcinogenesis, developing strategies that can allow its simultaneous use both in consolidated prevention protocols and in adequate treatment strategies, together with the solutions already solidly described in the literature.

Ultimately, after what has been described, it is possible to draw conclusions that consider berberine a transversal nutraceutical tool in metabolic disorders and breast cancer. These conclusions can be summarized in these points:

(1) Metabolic disorders (both those concerning the lipid and glucose profile of a patient) are recognized risk factors also for various types of cancer. In particular, given the profound relationship with the hormonal structure, many of the most important female neoplasms seem to be involved both in terms of incidence and progression and prognosis of the disease.
(2) Dysbiosis of the intestinal microbiota contributes to generating metabolic disorders, sub-clinical inflammation and, therefore, indirectly influencing the onset and progression of oncological diseases related to them.
(3) Berberine is certainly effective, both directly acting on the metabolic states and indirectly correcting and/or modulating the dysbiosis that generated them, ultimately working as a real anti-tumor compound both in a preventive sense (on risk factors) and a therapeutic one.

These important possibilities of use in the future may be integrated by bacterial therapy, when research can provide elements for a possible concrete clinical application. Together with the control of the weight, nutrition and lifestyle of patients, conventional therapies (metformin and statins in the first place) are certainly effective tools in controlling metabolic conditions.

From the above, it is no longer acceptable not to treat the metabolic conditions described and largely present in our patients. In particular, those metabolic conditions which are “nuanced” and “not striking” and which do not present immediate or evident clinical risk are too often underestimated. These conditions are not only a known cardiovascular risk factor but, as we have learned, also important elements that influence the onset and prognosis of many cancers, especially female ones. Too often, limiting oneself to advice on nutrition and lifestyle (however important) generates important clinical complications. Learning to identify and treat these conditions adequately and then learning to use all of the therapeutic tools available will inevitably result in advantages in terms of prevention and prognosis, which are ethically and clinically no longer negligible.

## Data Availability

Not applicable.
